# 1,4-Butandiol – Bestimmung von 1,4-Butandiol in der Luft am Arbeitsplatz mittels Gaschromatographie (GC-FID): Luftanalysen-Methode

**DOI:** 10.34865/am11063d10_1or

**Published:** 2025-03-31

**Authors:** Silke Werner, Lutz Nitschke, Ralph Hebisch, Andrea Hartwig, MAK Commission

**Affiliations:** 1 Institut für Arbeitsschutz der DGUV (IFA). Deutsche Gesetzliche Unfallversicherung e.V. (DGUV) Alte Heerstraße 111 53757 Sankt Augustin Deutschland; 2 Bayerisches Landesamt für Gesundheit und Lebensmittelsicherheit (LGL) Pfarrstraße 3 80538 München Deutschland; 3 Bundesanstalt für Arbeitsschutz und Arbeitsmedizin (BAuA) Friedrich-Henkel-Weg 1–25 44139 Dortmund Deutschland; 4 Institut für Angewandte Biowissenschaften. Abteilung Lebensmittelchemie und Toxikologie. Karlsruher Institut für Technologie (KIT) Adenauerring 20a, Geb. 50.41 76131 Karlsruhe Deutschland; 5 Ständige Senatskommission zur Prüfung gesundheitsschädlicher Arbeitsstoffe. Deutsche Forschungsgemeinschaft, Kennedyallee 40, 53175 Bonn, Deutschland. Weitere Informationen: Ständige Senatskommission zur Prüfung gesundheitsschädlicher Arbeitsstoffe | DFG

**Keywords:** 1,4-Butandiol, Luftanalysen, Analysenmethode, Arbeitsplatzmessung, Gefahrstoff, Gaschromatographie, Flammenionisationsdetektion, GC-FID, Glasfaserfilter, Aktivkohle

## Abstract

The working group “Air Analyses” of the German Senate Commission for the Investigation of Health Hazards of Chemical Compounds in the Work Area (MAK Commission) developed and verified the presented analytical method. It is used to determine the levels of 1,4-butanediol [110-63-44] in workplace air. The method covers concentrations in the range from one hundredth up to twice the current occupational exposure limit value (OELV) of 200 mg/m^3^. The method is also suitable for verifying the short-term exposure limit (STEL; excursion factor 4) for the inhalable fraction and vapour. Samples are collected by drawing a defined volume of air through a glass fibre filter and a sampling tube filled with activated charcoal which are inserted in a GGP mini sampling system using a flow regulated pump at a volumetric flow rate of 0.333 l/min. Exposure during the shift is assessed with a sampling period of 2 hours and the short-term exposure with a period of 15 minutes. The 1,4-butanediol deposited on the glass fibre filter and adsorbed to the activated charcoal is extracted by liquid desorption with dichloromethane/methanol (7:3 (v/v)) and analysed by gas chromatography using flame ionisation detection. The quantitative determination is based on multiple-point calibrations with an internal standard. A relative limit of quantification (LOQ) of 2 mg/m^3^ is obtained for an air sample volume of 40 litres. As the LOQ for a sample volume of 5 litres is below 400 mg/m^3^, the STEL can also be measured. The recovery is 95–101% and the expanded uncertainty is below 29% for a sampling period of 2 hours.

**Table:** 

**Methodennummer**	1
**Anwendbarkeit**	Luftanalyse
**Analyt. Messprinzip**	Gaschromatographie mit Flammenionisationsdetektion (GC-FID)

## 1 Kenndaten des Verfahrens

**Table:** 

**Präzision:**	Variationskoeffizient:	*V_x_* = 1,8 bis 6,5 %
	Erweiterte Messunsicherheit:	*U* = 28 %
	im Konzentrationsbereich von c = 2 bis 446 mg/m^3^ und n = 6 Bestimmungen	
**Bestimmungsgrenze:**	15,3 µg/ml
	2 mg/m^3^ bei einem Probeluftvolumen von 40 l und einer Probenahmedauer von 2 h	
**Wiederfindung:**	*η* = 0,95–1,01 (95–101 %)
**Probenahmeempfehlung:**	Probenahmedauer:	2 h
	Probeluftvolumen:	40 l
	Volumenstrom:	0,333 l/min
	Für Kurzzeitmessungen:	15 min, 0,333 l/min

## 2 Stoffbeschreibung

### 1,4-Butandiol [110-63-4]

**Abb. 1:**
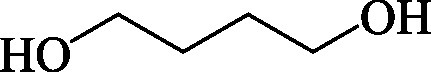
Strukturformel von 1,4-Butandiol

1,4-Butandiol (siehe Abbildung 1, auch Tetramethylenglykol, 1,4-Butylenglykol oder 1,4-Dihydroxybutan genannt) ist eine farblose, geruchlose Flüssigkeit (Molmasse 90,12 g/mol, Schmelzpunkt 20 °C, Siedepunkt 230 °C (IFA 2024), Dichte bei 25 °C 1,017 g/cm^3^ (Sigma-Aldrich 2024)). 1,4-Butandiol ist ein wichtiges Zwischenprodukt in der chemischen Industrie bei der Synthese anderer Substanzen, z. B. Tetrahydrofuran, und es dient als Ausgangsstoff für zahlreiche Folgeprodukte wie Polyester, Polyamide und Polyurethane. Darüber hinaus wird es als Lösungsmittel in Farben, Lacken und Tonern verwendet (ECHA 2024; RÖMPP-Redaktion 2017).

Für 1,4-Butandiol wurde ein Arbeitsplatzgrenzwert (AGW) von 200 mg/m^3^ und ein Überschreitungsfaktor von 4 festgelegt (AGS 2024). Ein MAK-Wert ist derzeit nicht aufgestellt (DFG 2024). Aufgrund der physikalisch-chemischen Eigenschaften kann 1,4-Butandiol sowohl dampf- als auch partikelförmig in der Luft am Arbeitsplatz vorliegen (DIN 2023).

## 3 Grundlage des Verfahrens

Das Analysenverfahren ermöglicht die Bestimmung des Gehaltes an 1,4-Butandiol in der Luft am Arbeitsplatz in einem Messbereich von 2 bis ca. 446 mg/m^3^. Dies entspricht einem Hundertstel bis dem Zweifachen des derzeit gültigen AGW.

Durch ein GGP-Mini, bestückt mit einem 13-mm-Glasfaserfilter und nachgeschaltetem Aktivkohleröhrchen (Typ BIA) wird mittels einer geeigneten Pumpe ein definiertes Luftvolumen gesaugt. In der Luft enthaltenes gasförmiges und partikuläres 1,4-Butandiol wird an den Probenträgern adsorbiert. Zur analytischen Bestimmung werden Filter und Aktivkohle gemeinsam mit Dichlormethan/Methanol (7:3 (V:V)) extrahiert. Die Trennung erfolgt gaschromatographisch auf einer polaren Trennsäule und die qualitative und quantitative Bestimmung mittels Flammenionisationsdetektion (GC-FID). Zur quantitativen Auswertung wird die Methode des internen Standards verwendet. 

## 4 Geräte, Chemikalien und Lösungen

### 4.1 Geräte

Für die Probenahme:

- Probenahmepumpe für personengetragene und stationäre Probenahme, geeignet für einen Volumenstrom von 0,333 l/min (z. B. GilAir Plus, Vertrieb durch DEHA Haan & Wittmer GmbH, 71296 Heimsheim)
- Personengetragener Probenahmekopf für die einatembare Fraktion (GGP-Mini-0,33) (z. B. Fa. GSA Messgerätebau GmbH, 40880 Ratingen)
- Glasfaserfilter, Ø 13 mm (z. B. MN 85/90 BF, Fa. Macherey und Nagel GmbH, 52355 Düren)
- Aktivkohle-Röhrchen, Typ BIA (z. B. Fa. Dräger AG & Co. KGaA, 23560 Lübeck)
- Glasschneider
- Silikonschlauch
- Silikon-Adapter GGP-Mini (z. B. Fa. Carmacon, 67574 Osthofen)
- Durchflussmesser (z. B. TSI Flowmeter 4146, Fa. TSI GmbH, 52068 Aachen)

Für die Probenaufbereitung und analytische Bestimmung:

- Schraubdeckelgläschen mit Deckel und Dichtplättchen, Nennvolumen 20 ml (z. B. Fa. LABC Labortechnik Zillger KG, 53773 Hennef)
- Einmalpinzetten (z. B. Fa. LABC Labortechnik Zillger KG, 53773 Hennef)
- Messkolben (Glas), Nennvolumen 5 ml (z. B. Fa. Brand GmbH + Co KG, 97877 Wertheim)
- Braunglasflasche 1000 ml (z. B. Fa. Brand GmbH + Co KG, 97877 Wertheim)
- Messzylinder 1000 ml (z. B. Fa. Brand GmbH + Co KG, 97877 Wertheim)
- Variable Kolbenhubpipetten 10–100 µl und 100–1000 µl mit 100-, 200- und 1000-µl-Pipettenspitzen (z. B. Eppendorf Multipette E3 mit Combitips, Fa. Eppendorf, 22366 Hamburg)
- Mikroliterspritzen, Nennvolumen 1 µl (z. B. Fa. Hamilton Germany GmbH, 82166 Gräfelfing)
- Gaschromatograph mit Flammenionisationsdetektor (FID) und Datenauswerteeinheit (z. B. Fa. PerkinElmer LAS GmbH, 63110 Rodgau)
- Einmalspritzen, Volumen 2 ml mit Einmalkanülen 0,9 × 40 mm (z. B. Fa. B. Braun SE, 34212 Melsungen)
- Spritzenvorsatzfilter mit Polytetrafluorethylen-Membran (PTFE) 13 mm, Porengröße 0,45 µm (z. B. VWR, 64293 Darmstadt)
- Autosamplergläschen, Nennvolumen ca. 1,5 ml (z. B. LABC Labortechnik Zillger KG, 53773 Hennef)
- Schraubkappen für Autosamplergläschen (z. B. CS-Chromatographie-Service GmbH, 52379 Langerwehe)
- Analysenwaage, Wägebereich von 0,01 mg bis 220 g, Ablesbarkeit 0,01 mg (z. B. XP 205 Delta Range, Fa. Mettler-Toledo GmbH, 35396 Gießen)
- Polare Trennsäule (z. B. StabilWax, Länge (L) 60 m, Innendurchmesser (ID) 0,25 mm, Filmdicke (FD) 0,5 µm, Fa. Restek GmbH, 61348 Bad Homburg)

### 4.2 Chemikalien

- Methanol zur Analyse, ≥ 99,9 % (z. B. Fa. Merck KGaA, 64293 Darmstadt)
- Dichlormethan zur Analyse, ≥ 99,8 % (z. B. Fa. Merck KGaA, 64293 Darmstadt)
- 1,4-Butandiol, 99 % (z. B. ReagentPlus, Fa. Sigma-Aldrich Chemie GmbH, 82024 Taufkirchen)
- 1-Hexanol zur Synthese, ≥ 98,0 % (z. B. Fa. Merck KGaA, 64293 Darmstadt)
- Helium 5.0
- Wasserstoff 5.0
- Synthetische Luft (kohlenwasserstofffrei)

### 4.3 Lösungen

Unter Verwendung der in Abschnitt 4.2 aufgeführten Chemikalien werden folgende Lösungen hergestellt:

**Extraktionslösung: **(Dichlormethan/Methanol, 7:3 (V:V))

Zur Herstellung der Extraktionslösung werden einzeln und nacheinander in einem 1000-ml-Messzylinder 700 ml Dichlormethan und 300 ml Methanol abgemessen und in eine geeignete Braunglasflasche überführt. Die Flasche wird verschlossen und geschüttelt. 

Das Lösemittelgemisch kann bei Raumtemperatur gelagert werden und ist 4 Wochen haltbar.

**Stammlösung Kalibrierung:**(19,3 mg 1,4-Butandiol/ml)

In einem 5-ml-Messkolben werden ca. 3 ml Extraktionslösung vorgelegt. Anschließend werden mit einer Multipette 95 µl 1,4-Butandiol in den Messkolben dosiert. Der Messkolben wird mit Extraktionslösung bis zur Messmarke aufgefüllt und geschüttelt.

Die Stammlösung Kalibrierung wird in ein passendes Schraubdeckelgläschen abgefüllt, mit einem Schraubdeckel verschlossen, beschriftet und mit dem Datum des Ansetzens versehen. Sie ist bei 6 °C im Kühlschrank mindestens sechs Monate haltbar. 

**Stammlösung Kontrolle:** (4,1 mg 1,4-Butandiol/ml)

In einem 5-ml-Messkolben werden ca. 3 ml Extraktionslösung vorgelegt. Anschließend werden mit einer Multipette 20 µl 1,4-Butandiol in den Messkolben dosiert. Der Messkolben wird mit Extraktionslösung bis zur Messmarke aufgefüllt und geschüttelt.

Die Stammlösung Kontrolle wird unabhängig von der Stammlösung Kalibrierung angesetzt.

Die Stammlösung Kontrolle wird in ein passendes Schraubdeckelgläschen abgefüllt, mit einem Schraubdeckel verschlossen, beschriftet und mit dem Datum des Ansetzens versehen. Sie ist bei 6 °C im Kühlschrank mindestens sechs Monate haltbar. 

### 4.4 Kalibrierstandards

Ausgehend von der Stammlösung Kalibrierung werden durch Verdünnung fünf Kalibrierlösungen hergestellt. 

In je einem 5-ml-Messkolben werden ca. 1,5 ml Extraktionslösung vorgelegt. Es werden die in Tabelle 1 ange­gebenen Volumina der Stammlösung Kalibrierung zudosiert und danach der Messkolben bis zur Messmarke mit Extraktionslösung aufgefüllt. Anschließend werden 0,5 μl 1-Hexanol als interner Standard (ISTD) mit einer entsprechenden Mikroliterspritze zugegeben und geschüttelt.

Die Kalibrierlösungen sind vor jeder Kalibrierung frisch anzusetzen.

**Tab. 1:** Herstellung und Konzentrationen der Kalibrierstandards

Volumen der Stammlösung Kalibrierung [µl]	Massenkonzentration 1,4-Butandiol [µg/ml]
4,0	15,4
20	77,3
40	155
70	271
150	580

### 4.5 Kontrollstandards

Arbeitstäglich sind Kontrollstandards in Doppelbestimmung unter den in Abschnitt 6 angegebenen Bedingungen zu analysieren, deren Konzentration im unteren Arbeitsbereich liegt.

**Kontrollstandard: **(21,2 µg 1,4-Butandiol/ml)

In einen 5-ml-Messkolben, in dem ca. 1,5 ml Extraktionslösung vorgelegt wurden, werden mit der Multipette 26 µl Stammlösung Kontrolle überführt, mit Extraktionslösung bis zur Marke aufgefüllt, mit 0,5 µl ISTD versetzt und geschüttelt. Die Lösung wird in Autosamplergläschen abgefüllt und für die arbeitstägliche Überprüfung im Kühlschrank aufbewahrt. Bei einer Abweichung der Konzentration von mehr als ± 10 %, ist ein frischer Kontrollstandard anzusetzen, mit dem die Kontrolle dann zu wiederholen ist.

## 5 Probenahme und Probenaufbereitung

### 5.1 Probenahme

Die Probenahme kann sowohl ortsfest als auch personengetragen erfolgen. Bei personengetragener Probenahme erfolgt diese im Atembereich. Es ist darauf zu achten, dass die Öffnung des Sammelkopfes frei zugänglich ist.

Für die Probenahme werden geeignete durchflussstabilisierte Pumpen eingesetzt. Mithilfe eines repräsentativen Pro­benträgers als Vorwiderstand wird der Volumenstrom auf 20 l/h (0,333 l/min) eingestellt.

Unmittelbar vor der Probenahme wird das Aktivkohleröhrchen mit einem Glasschneider beidseitig geöffnet. Das Röhrchen wird mit einem Schlauchstück (Silikon) unter Beachtung der vorgesehenen Flussrichtung an der einen Seite mit dem Probenahmekopf, der den Glasfaserfilter enthält, verbunden. Das andere Ende des Aktivkohleröhrchens wird in Pfeilrichtung mit der Pumpe verbunden. Die empfohlene Probenahmedauer beträgt zwei Stunden. Bei einer zweistündigen Probenahme mit einem Volumenstrom von 0,333 l/min entspricht dies einem Probeluftvolumen von 40 l. Die für die Bestimmung der Luftkonzentration wichtigen Parameter (Probeluftvolumen, Temperatur, Luftdruck und relative Luftfeuchte) werden im Probenahmeprotokoll vermerkt.

Unmittelbar nach der Probenahme wird das Aktivkohleröhrchen mit den dafür vorgesehenen Kappen verschlossen. Der Probenahmekopf des GGP-Mini wird aufgeschraubt. Der Glasfaserfilter wird mithilfe einer Pinzette in das mitgeführte Schraubdeckelgläschen überführt und dieses anschließend luftdicht verschlossen. Die beaufschlagten Probenträger sind dem Analysenlabor verschlossen möglichst innerhalb von 7 Tagen zuzuleiten und werden dort bis zur Aufarbeitung bei Raumtemperatur gelagert.

Nach der Probenahme ist der Volumenstrom auf Konstanz zu überprüfen. Ist die Abweichung vom eingestellten Volumenstrom ≥ ± 5 %, wird empfohlen, die Messung zu wiederholen.

### 5.2 Probenaufbereitung

Das Aktivkohleröhrchen wird im Labor geöffnet und der Inhalt vollständig in das Schraubdeckelgläschen mit dem zugehörigen Glasfaserfilter überführt. Anschließend werden Glasfaserfilter und Aktivkohle mit 5 ml Extraktionslösung überschichtet und 0,5 µl 1-Hexanol als ISTD zudosiert. Das Gläschen wird verschlossen und über Nacht bei Raumtemperatur stehen gelassen. Der Extrakt wird anschließend mit einer Einmalspritze aufgenommen und durch einen Spritzenvorsatzfilter in ein Autosamplergläschen filtriert. Die Probe wird unter den in Abschnitt 6 genannten Bedingungen in Doppelbestimmung gaschromatographisch analysiert. Sollte das erhaltene Signal außerhalb des Messbereichs liegen, wird die Probe entsprechend verdünnt und erneut analysiert.

## 6 Instrumentelle Arbeitsbedingungen

**Table:** 

**Gerät:**	Gaschromatograph PerkinElmer 500, mit FID und automatischem Probengeber	
**Trennsäule:**	Material: Länge: Innendurchmesser: Filmdicke:	StabilWax 60 m 0,25 mm 0,5 µm
**Injektionsvolumen:**	1 µl	
**Injektortemperatur:**	200 °C	
**Detektor:**	FID	
**Detektortemperatur:**	250 °C
**Ofenprogramm:**	Anfangstemperatur 50 °C; 2,5 min halten 20 °C/min auf 200 °C aufheizen; 10 min halten	
**Trägergas:**	Helium 5.0
**Trägergasstrom:**	280 kPa
**Split:**	15 ml/min

## 7 Analytische Bestimmung

Mit Hilfe eines Autosamplers werden zweimal jeweils 1 μl der aufgearbeiteten Probe in den Gaschromatographen injiziert und unter den in Abschnitt 6 angegebenen Bedingungen analysiert.

Liegen die ermittelten Konzentrationen oberhalb des Kalibrierbereiches, wird von der Messprobe eine geeignete Verdünnung in Extraktionslösung angesetzt und diese nochmals analysiert.

## 8 Kalibrierung

Zur Erstellung der Kalibrierfunktionen werden die in Abschnitt 4.4 beschriebenen Kalibrierstandards verwendet. Von den Kalibrierstandards werden dreimal jeweils 1 μl injiziert und entsprechend den Abschnitten 6 und 7 analy­siert. Der Quotient der ermittelten Peakflächen von 1,4-Butandiol und ISTD werden gegen den Quotienten der jewei­ligen Konzentrationen von 1,4-Butandiol und ISTD aufgetragen. Die Kalibrierfunktion ist über den untersuchten Konzentrationsbereich linear.

Zur Überprüfung der Kalibrierfunktion sind arbeitstäglich Kontrollstandards zu analysieren (siehe Abschnitt 4.5). Die Kalibrierung ist neu zu erstellen, wenn die analytischen Bedingungen sich ändern oder die Qualitätskontrolle dazu Anlass gibt.

## 9 Berechnung des Analysenergebnisses

Die Berechnung der Konzentration in der Extraktionslösung erfolgt mithilfe der von der Datenauswerteeinheit ermit­telten Masse an 1,4-Butandiol pro Probenträger nach Gleichung 1. 


(1)

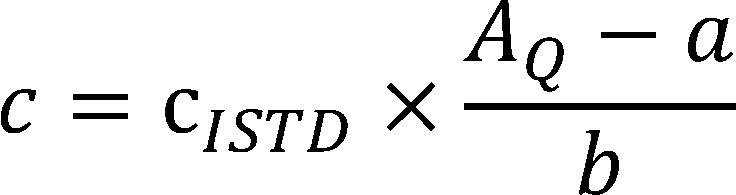



Es bedeuten: 

**Table:** 

*c*	Massenkonzentration der Substanz in der Extraktionslösung in mg/l
*c_ISTD_*	Massenkonzentration des ISTD in der Extraktionslösung in mg/l
*A_Q_*	Flächenquotient
*a*	Ordinatenabschnitt
*b*	Steigung des Graphen

Daraus wird unter Berücksichtigung des Extraktionsvolumens, des Probeluftvolumens und der Wiederfindung die Konzentration in der Luft im Arbeitsbereich nach Gleichung 2 berechnet: 


(2)

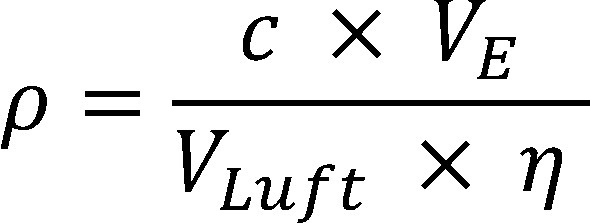



**Table:** 

*ρ*	Massenkonzentration der Substanz in der Luftprobe in mg/m^3^
*V_E_*	Volumen der Extraktionslösung in l (hier 0,005 l)
*V_Luft_*	Probeluftvolumen in m^3^ (ermittelt aus Volumenstrom und Probenahmedauer, hier bei 2-stündiger Probenahmedauer 0,04 m^3^)
*ƞ*	Wiederfindung

## 10 Beurteilung des Verfahrens

Die Kenndaten der Methode wurden nach Maßgabe der DIN EN 482 (DIN 2021), ISO 20581 (DIN 2016), DIN 32654 (DIN 2008) und DIN EN ISO 23861 (DIN 2023) ermittelt.

Zur Ermittlung der Verfahrenskenndaten wurden die Probenträger, wie unter Abschnitt 5.1 beschrieben, vorbereitet. Die Glasfaserfilter wurden jeweils mit entsprechenden Lösungen von 1,4-Butandiol dotiert. Die beaufschlagten Mengen an 1,4-Butandiol lagen im Bereich von 0,08 mg bis 17,8 mg pro Probenträger. Dies entspricht unter der Annahme von 40 l Probeluftvolumen einem Konzentrationsbereich von 2,0 mg/m^3^ bis 446 mg/m^3^. Durch die dotierten Probenträger wurde für 2 Stunden konditionierte Luft mit einem Volumenstrom von 0,333 l/min aus einer dynamischen Prüfgasstrecke gesaugt.

### 10.1 Präzision, Wiederfindung und erweiterte Messunsicherheit

Die Versuche zur Präzision und Wiederfindung wurden bei einer relativen Luftfeuchte von ca. 40 % durchgeführt. Die Analyse der Proben erfolgte wie in den Abschnitten 5 und 6 beschrieben. 

Im Rahmen der Bestimmung der Wiederfindung wurden bei jedem Versuch sechs Probenträger belegt, die daraus ermit­telten Variationskoeffizienten und Wiederfindungen sowie die daraus berechneten erweiterten Messunsicherheiten können Tabelle 2 entnommen werden. Die Berechnung erfolgte nach Vorgaben der DIN EN ISO 23861 (DIN 2023) unter Verwendung der IFA-Software – Berechnung der erweiterten Messunsicherheit (IFA o.J.).

**Tab. 2:** Verfahrenskenndaten und erweiterte Messunsicherheit

Konzentration^a)^ [mg/m^3^]	Wiederfindung [%]	Variationskoeffizient [%]	Erweiterte Messunsicherheit *U* [%]
2,0	95	5,1	28,3
20	100	6,5	28,3
91	98	6,5	28,4
202	100	4,7	28,4
446	100	1,8	27,9

^a)^ Die Konzentration ergibt sich für eine zweistündige Probenahme bei einem Volumenstrom von 0,333 l/min.

Die erweiterte Messunsicherheit wurde unter Abschätzung aller relevanten Einflussgrößen ermittelt. Die Ergebnis­unsicherheit umfasst zwei wesentliche Beiträge, die Unsicherheitskomponenten der Probenahme und der Analyse.

Die Kombination aller Unsicherheitsbeiträge führt zu konzentrationsabhängigen kombinierten Messunsicherheit des Gesamtverfahrens. Durch Multiplikation mit dem Erweiterungsfaktor k = 2 erhält man die in Tabelle 2 angegebenen Werte der erweiterten Messunsicherheit für das Gesamtverfahren.

### 10.2 Bestimmungsgrenze

Die Bestimmungsgrenze (BG) wurde nach der Leerwertmethode gemäß DIN 32654 (DIN 2008) ermittelt. Die Bestimmungsgrenzen betragen 15,3 µg/ml absolut oder 2 mg/m^3^ für ein Probenluftvolumen von 40 Litern (0,333 l/min und 2 h Probenahme).

### 10.3 Einfluss der relativen Luftfeuchte

Es wurden Probenahmeversuche bei ca. 20 % und ca. 80 % relativer Luftfeuchte durchgeführt. Für jede der untersuchten Konzentrationen (BG, 0,1 AGW, 1 AGW und 2 AGW) wurden je sieben Probenträger, wie unter Abschnitt 10 beschrieben, beaufschlagt.

Im Bereich der Bestimmungsgrenze konnte bei einer Luftfeuchte von ca. 20 % ein Einfluss der Luftfeuchte auf die Wiederfindung festgestellt werden. Im Bereich der Bestimmungsgrenze beträgt sie 94,6 %. In diesem Fall muss der Messwert entsprechend eingeschränkt bzw. korrigiert werden.

Der Einfluss der Luftfeuchte für den Bereich zwischen 0,01 AGW und 0,1 AGW muss gesondert untersucht werden. Bei Konzentrationen von 0,1 AGW bis 2 AGW konnte kein Einfluss der Luftfeuchte auf die Wiederfindung festgestellt werden. 

### 10.4 Einfluss der Temperatur

Zur Überprüfung des Temperatureinflusses wurden Probenahmeversuche bei Temperaturen von ca. 10 °C und ca. 40 °C durchgeführt. Dazu wurden je untersuchter Konzentration (BG, 0,1 AGW und 2 AGW) sieben Probenträger, wie unter Abschnitt 10 beschrieben, vorbereitet. Die Probenträger wurden in einem Klimaschrank gelagert und währenddessen mittels einer geeigneten Probenahmepumpe für zwei Stunden Luft mit einem Volumenstrom von 0,333 l/min durch den Probenträger gesaugt.

Im untersuchten Konzentrationsbereich konnte an der Bestimmungsgrenze, bei einer Temperatur von ca. 10 °C, ein Einfluss auf die Wiederfindung festgestellt werden. Diese beträgt bei den genannten Bedingungen 72 %. Der Messwert muss in diesem Fall entsprechend korrigiert werden. Bei Konzentrationen von 0,1 AGW bis 2 AGW konnte kein Einfluss der Temperatur auf die Wiederfindung festgestellt werden. 

### 10.5 Kapazität des Probenahmesystems

Zur Bestimmung des Durchbruchverhaltens des eingesetzten Probenahmesystems wurde das mit einem Glasfaserfilter und einem Aktivkohleröhrchen bestückte GGP-Mini mittels eines Schlauches mit einem weiteren Aktivkohleröhrchen verbunden. Es wurden 25,9 mg 1,4-Butandiol auf den Glasfaserfilter dotiert, was bei einer dreistündigen Probenahme einer Luftkonzentration von 433 mg/m^3^ entspricht. Anschließend wurde für drei Stunden (empfohlene Probenahme­dauer plus eine Stunde) konditionierte Luft (0,333 l/min) aus einer dynamischen Prüfgasstrecke durch die Anordnung gesaugt. Danach wurden jeweils der Filter und der Inhalt des ersten Aktivkohleröhrchen zusammen und der Inhalt des zweiten Röhrchens getrennt in je ein Schraubdeckelgläschen überführt und, wie in den Abschnitten 5.2, 6 und 7 beschrieben, aufgearbeitet und analysiert.

Die Wiederfindung des auf dem Glasfaserfilter und dem ersten Aktivkohleröhrchen adsorbierten 1,4-Butandiol lag bei 99 %. Auf dem nachgeschalteten Röhrchen wurde kein 1,4-Butandiol nachgewiesen. Somit ist das Probenahmesystem auch für Konzentrationen bis 433 mg/m^3^ bei einer dreistündigen Probenahme geeignet.

### 10.6 Lagerfähigkeit

Zur Ermittlung der Lagerfähigkeit beaufschlagter Probenträger wurden jeweils sechs Probenträger, wie unter Abschnitt 10 beschrieben, mit Konzentrationen von 2,0 mg/m^3^, 20,3 mg/m^3^ und 406 mg/m^3^ dotiert und für 2 Stunden Luft mit einem Volumenstrom von 0,333 l/min bei hoher Luftfeuchte (ca. 80 %) konditioniert. Die Filter wurden mit einer Pinzette dem GGP-Mini entnommen, in ein Schraubdeckelgläschen überführt und dieses verschlossen bei Raumtemperatur gelagert. Die Aktivkohleröhrchen wurden mit den Kappen verschlossen und ebenfalls bei Raumtemperatur gelagert. Anschließend wurden die Filter und Sammelröhrchen gemäß den Abschnitten 5.2, 6 und 7 nach einem und drei Tagen sowie nach einer, zwei, drei und vier Wochen Lagerung aufgearbeitet und analysiert.

Die Lagerfähigkeit der beaufschlagten Probenträger beträgt maximal 7 Tage.

### 10.7 Selektivität

Alle verwendeten Materialien und Reagenzien sind auf Blindwerte insbesondere bei Chargenwechsel zu überprüfen. Bei der Verfahrensausarbeitung wurde kein Blindwert festgestellt, sodass für diesen Fall nicht korrigiert werden musste. 

**Abb. 2:**
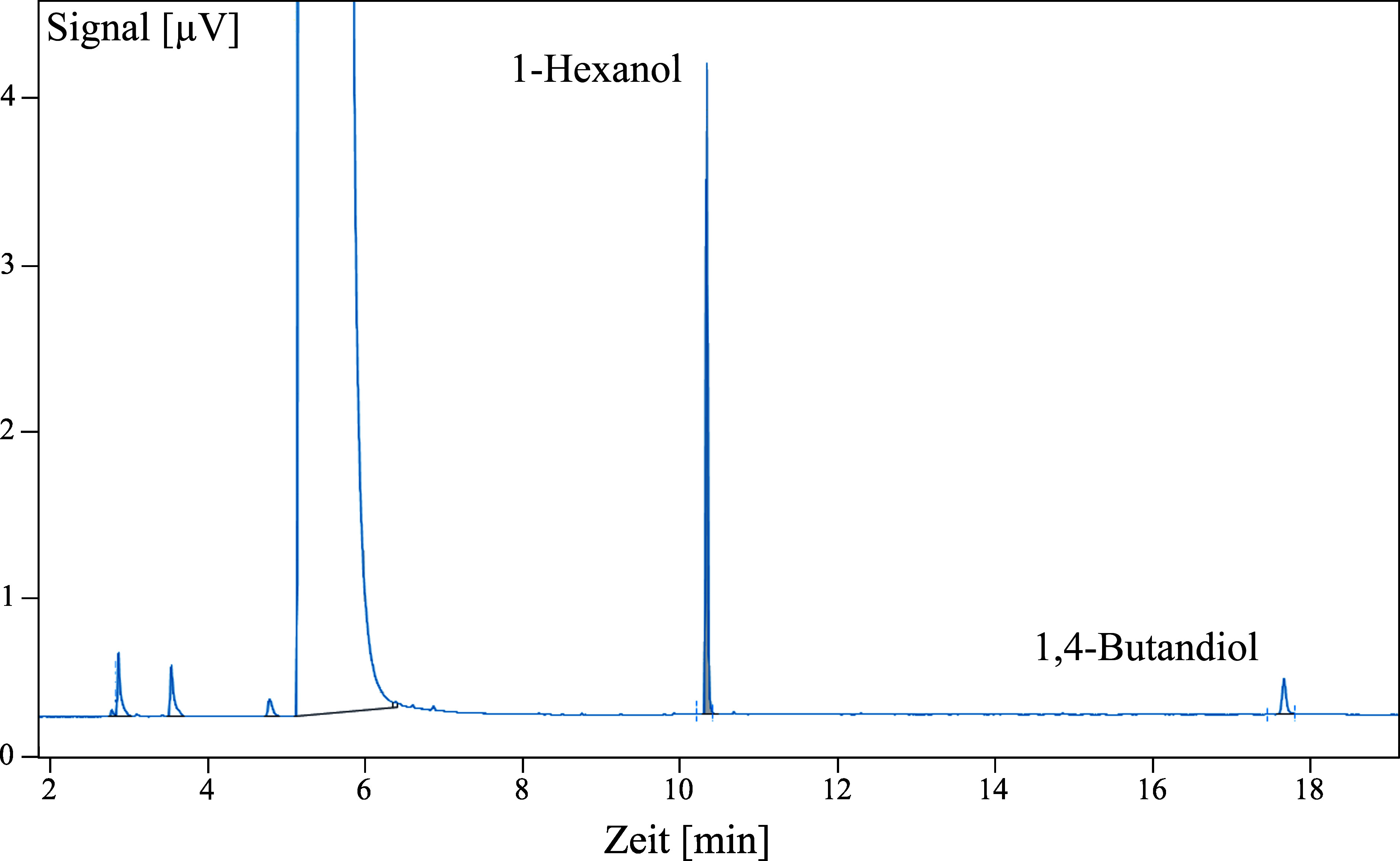
Gaschromatogramm von 1,4-Butandiol mit internem Standard 1-Hexanol

Unter den angegebenen Bedingungen ist die Bestimmung von 1,4-Butandiol selektiv. Die Signale des Analyten und ISTD sind gut getrennt (siehe Abbildung 2). Sollte dennoch ein Signal gleicher Retentionszeit wie die des Analyten bzw. des ISTD vorhanden sein, muss dieses genauer untersucht und der Blindwert bei der Analysenberechnung der Proben berücksichtigt werden.

## 11 Diskussion

Das beschriebene Messverfahren ermöglicht die Bestimmung von 1,4-Butandiol in der Luft am Arbeitsplatz in einem Konzentrationsbereich von einem Hundertstel bis zum Doppelten des derzeit gültigen AGW von 200 mg/m^3^. Das Messverfahren ist auch geeignet, um die Einhaltung des Kurzzeitwertes zu überprüfen.
